# Supplementary material for: Arabica coffee Intercropped with Urochloa decumbens Improved Nutrient Uptake and Yield in the Brazilian Cerrado
Source: Plants (Basel). 2025 Feb 7;14(4):496. doi: 10.3390/plants14040496 (PMC11860056; doi:10.3390/plants14040496)
Supplement: Supplementary file 1 [file plants-14-00496-s001.zip › plants-3392053-supplementary.pdf]

**Macronutrients**	**Equation**
N	IPR103 WB: $y=2.877e^{+1} +2.964e^{-2} *x -1.954e^{-4} *x^2(R^2=0.66^*)$
	IPR99 WB: $y=2.613e^{+1} +5.925e^{-2} *x -2.670e^{-4} *x^2(R^2=0.68^*)$
	IPR 103 NB: $y=1.736e^{+1} +1.653e^{-1} *x -4.784e^{-4} *x^2(R^2=0.63^{ns})$
	IPR99 NB: $y=2.625e^{+1} +7.889e^{-2} *x -3.217e^{-4} *x^2(R^2=0.70^*)$
P	IPR103 WB: $y=1.054e^{+1} -9.376e^{-2} *x +2.794e^{-4} *x^2(R^2=0.81^*)$
	IPR99 WB: $y=1.050e^{+1} -1.032e^{-1} *x +3.256e^{-4} *x^2(R^2=0.82^*)$
	IPR 103 NB: $y=1.002e^{+1} -8.772e^{-2} *x +2.883e^{-4} *x^2(R^2=0.76^*)$
	IPR99 NB: $y=1.105e^{+1} -1.019e^{-1} *x +3.288e^{-4} *x^2(R^2=0.78^*)$
K	IPR103 WB: $y=4.207e^{+0}\ 7.927e^{-3} *x -1.840e^{-5} *x^2(R^2=0.09^{ns})$
	IPR99 WB: $y=3.286e^{+0}\ 2.181e^{-2} *x -5.319e^{-5} *x^2(R^2=0.031^{ns})$
	IPR 103 NB: $y=4.730e^{+0} +1.002e^{-2} *x -3.080e^{-5} *x^2(R^2=0.11^{ns})$
	IPR99 NB: $y=6.941e^{+0} -5.607e^{-3} *x +1.494e^{-5} *x^2(R^2=0.04^{ns})$
Mg	IPR103 WB: $y=5.774e^{+0} -1.730e^{-2} *x +4.175e^{-5} *x^2(R^2=0.27^{ns})$
	IPR99 WB: $y=5.296e^{+0} -1.196e^{-2} *x +2.700e^{-5} *x^2(R^2=0.17^{ns})$
	IPR 103 NB: $y=4.982e^{+0} -7.378e^{-3} *x +1.625e^{-5} *x^2(R^2=0.10^{ns})$
	IPR99 NB: $y=3.800e^{+0} +4.683e^{-3} *x -1.821e^{-5} *x^2(R^2=0.13^{ns})$
Ca	IPR103 WB: $y=1.187e^{+1} -3.787e^{-2} *x+8.980e^{-5} *x^2(R^2=0.44^*)$
	IPR99 WB: $y= 1.189e^{+1} -4.925e^{-2} *x +1.212e^{-4} *x^2(R^2=0.33^*)$
	IPR 103 NB: $y=1.256e^{+1} -5.358e^{-2} * x+ 1.258e^{-4} *x^2(R^2=0.33^*)$
	IPR99 NB: $y=1.187e^{+1} -3.787e^{-2} *x + 8.980e^{-5} *x^2(R^2=0.22^*)$
S	IPR103 WB: $y=2.594e^{+0} -9.316e^{-3} *x +2.291e^{-5} *x^2(R^2=0.64^{ns})$
	IPR99 WB: $y=2.232e^{+0} -6.68e^{-3} *x +1.900e^{-5} *x^2(R^2=0.40^{ns})$
	IPR 103 NB: $y=2.749e^{+0} -1.043e^{-2} *x +2.638e^{-5} *x^2(R^2=0.53^{ns})$
	IPR99 NB: $y=2.786e^{+0} -1.120e^{-2} *x +2.963e^{-5} *x^2(R^2=0.59^{ns})$
**Micronutrients**	**Equation**
Cu	IPR103 WB: $y=3.388e^{+0} +5.946e^{-2} *x -2.185e^{-4} *x^2(R^2=0.65^*)$
	IPR99 WB: $y=3.082e^{+0} +5.705e^{-2} *x -2.093e^{-4} *x^2(R^2=0.69^*)$
	IPR103 NB: $y=2.269e^{+0} +3.556e^{-2} *x -1.320e^{-4} *x^2(R^2=0.63^{ns})$
	IPR99 NB: $y=2.269e^{+0} +3.556e^{-2} *x -1.320e^{-4} *x^2(R^2=0.63^{ns})$
Fe	IPR103 WB: $y=1.450e^{+2} +5.076e^{-1} *x -2.806e^{-3} *x^2(R^2=0.43^{ns})$
	IPR99 WB: $y=1.017e^{+2} +5.526e^{-1} *x -2.508e^{-3} *x^2(R^2=0.43^{ns})$
	IPR 103 NB: $y=1.217^{+2} +4.605e^{-1} *x -2.502e^{-5} *x^2(R^2=0.45^{ns})$
	IPR99 NB: $y=1.290e^{+2} +4.722e^{-1} *x -2.610e^{-3} *x^2(R^2=0.45^{ns})$
Mn	IPR103 WB: $y=8.379e^{+1} -3.962e^{-1} *x +8.331e^{-4} *x^2(R^2=0.65^{ns})$
	IPR99 WB: $y=7.238e^{+1} -3.077e^{-1} *x +6.322e^{-4} *x^2(R^2=0.54^{ns})$
	IPR 103 NB: $y=5.899e^{+1} -1.072e^{-1} *x +3.669e^{-5} *x^2(R^2=0.53^{ns})$
	IPR99 NB: $y=6.006e^{+1} -1.833e^{-1} *x +2.710e^{-4} *x^2(R^2=0.64^{ns})$
B	IPR103 WB: $y=4.890e^{+1} +2.058e^{-1} *x -1.119e^{-3} *x^2(R^2=0.57^*)$
	IPR99 WB: $y=4.776e^{+1} +2.343e^{-1} *x -1.178e^{-3} *x^2(R^2=0.59^*)$
	IPR 103 NB: $y=4.890e^{+1} +2.058e^{-1} *x -1.119e^{-3} *x^2(R^2=0.58^*)$
	IPR99 NB: $y=5.038e^{+1} +2.154e^{-1} *x -1.161e^{-3} *x^2(R^2=0.57^*)$
